# Low Stroke Risk in Children With Sickle Cell Disease in French Guiana: A Retrospective Cohort Study

**DOI:** 10.3389/fmed.2022.851918

**Published:** 2022-06-28

**Authors:** Julie Gargot, Marie-Claire Parriault, Antoine Adenis, Jérôme Clouzeau, Kim-Anh Dinh Van, Balthazar Ntab, Antoine Defo, Mathieu Nacher, Narcisse Elenga

**Affiliations:** ^1^Service de Pédiatrie, Centre Hospitalier Andrée Rosemon, Cayenne, French Guiana; ^2^CIC INSERM 1424, Centre Hospitalier Andrée Rosemon, Cayenne, French Guiana; ^3^Service de Pédiatrie, Centre Hospitalier de l’Ouest-Guyanais «Franck Joly», Saint-Laurent-du-Maroni, French Guiana; ^4^Département d’Information Médical, Centre Hospitalier de Kourou, Kourou, French Guiana; ^5^Département d’Information Médicale, Centre Hospitalier de l’Ouest-Guyanais «Franck Joly», Saint-Laurent-du-Maroni, French Guiana

**Keywords:** sickle cell disease, stroke, incidence, French Guiana, children

## Abstract

One in every 227 babies born in French Guiana has sickle cell disease, which represents the greatest incidence in France. This study aimed to determine the incidence of stroke in children with sickle cell disease and its associated risk factors. This retrospective cohort study included all children with sickle cell disease diagnosed in the neonatal period who were born in French Guiana between 01/01/1992 and 12/31/2002. Of a total of 218 records, 122 patients were included. There were 70 HbSS/Sβ0 (58%), 40 HbSC (33%), and 11 Sβ + thalassemia (9%). The number of emergency admissions was significantly different between genotypes, with a higher number in SS/Sβ0 children (*p* = 0.004). There were significantly more acute chest syndromes (*p* = 0.006) and more elevated Lactate Dehydrogenase in SS/Sβ0 patients (*p* = 0.003). Three of these patients had ischemic strokes at a mean age of 6.9 years, and one had a hemorrhagic stroke at the age of 9,2 years. The incidence rate of ischemic stroke for SS/Sβ0 children was 3.1 (95% CI: 1.0–9.7) per 1,000 patient-years, and the clinically apparent stroke risk by the age of 15 years and 3 months was 6,4%. The incidence of hemorrhagic stroke was 1.1 (95% CI: 0.1–7.4) per 1,000 patients-years. No patient with SC or Sβ + thalassemia genotypes experienced any stroke.

## Introduction

Sickle cell disease (SCD) is an autosomal recessive inherited disease characterized by the synthesis of an abnormal hemoglobin sickle hemoglobin S (βs, HbS) resulting in the substitution of a single amino acid (Glu→Val) at the sixth position of β-chain of normal hemoglobin (HbA) molecule (1, 2). This point mutation leads to the polymerization of the HbS molecule under deoxygenated conditions. Polymerization of the HbS leads to the stiffening and weakening of sickled red blood cells. This hereditary disease particularly affects those of African origin because the protection against malaria that heterozygocy conferred to their ancestors selected the sickle cell mutations genes (3). Homozygous SS (sickle cell anemia or SCA) is generally considered to be the most severe form of SCD. Compound heterozygotes, in whom HbS is combined with a different mutation of the second β-globin gene such as HbC, D, OArab or β-thalassemia (where synthesis of β-globin is reduced), are also considered as symptomatic forms of SCD, with varying phenotypes. SCD is characterized by abnormally shaped adhesive red blood cells that interact with white blood cells and the vascular endothelium, resulting in chronic hemolysis, reperfusion injury, vasculopathy impaired vasomotor tone, and a prothrombotic state. SCD brain vasculopathy causes both overt strokes and “silent” cerebral infarcts, resulting in early death, permanent neurocognitive and physical dysfunction together with life-long suffering and poor quality of life. Pathophysiology of stroke in SCD is complex and includes both diseased large arteries and penetrating arteries (4). SCD is the most common cause of pediatric stroke and children with SCA are at higher risk of stroke, notably in the first decade of life (3). In the absence of early Transcranial Doppler (TCD) screening, strokes occur in 7.4–11% of patients with SCA before age 20 (5–7). As the risk of stroke persists throughout childhood (8), TCD screening has been implemented to detect SCA children at risk, in order to initiate transfusion programs known for significantly reducing stroke incidence in SCA patients with an abnormal TCD (9–12).

French Guiana is an overseas French territory on the north-eastern coast of South America. SCD is a major public health problem in French Guiana (13). The population, most of which is of African ancestry, consists mainly of three groups: Guianese Creoles, Maroons (descendants of runaway slaves), and, more recently, Haitian immigrants (14). The estimated incidence of SCD at birth is one in 227, and the overall frequency of hemoglobin AS carriers is 10% (13, 15). The major SCD groups include the three main genetic forms that combine different structural hemoglobin variants or thalassemia syndromes (hemoglobin S HbS, hemoglobin C HbC, β-thalassemia) (16). There are scarce data on the actual incidence of SCA-related strokes in French Guiana. We aimed to determine the risk for stroke in children with SCD and the risk factors associated with stroke.

## Materials and Methods

### Stroke Definition

Stroke is classically defined as a neurological deficit related to an acute focal injury to the central nervous system (CNS) of vascular origin, including cerebral infarction, intracerebral hemorrhage (ICH), or subarachnoid hemorrhage (SAH) (17). Due to significant advances in the nature, timing, clinical recognition of stroke, its differential diagnoses, and imaging results, authors called for an update of definition of stroke (18).

### Definition of Silent Cerebral Infarction

Silent cerebral infarction (SCI) is defined as an area of abnormal hyperintensity on T2-weighted (axial and coronal) MRI images of the brain in a patient without a history of neurological symptoms or signs consistent with the location of the infarct in a given vascular distribution.

### Definition of Other Conditions

Acute chest syndrome (ACS) is defined by the presence of fever and/or respiratory symptoms, accompanied by a new radiodensity on the chest X-ray.

Asthma is a chronic inflammatory lung disease, associated with airway hyper responsiveness that leads to recurrent episodes of wheezing, breathlessness, chest tightness and coughing.

Adenoid hypertrophy is an obstructive disorder related to an increase in the size of the adenoids (a collection of lymphoepithelial tissues located in the upper part of the nasopharynx, medial to the openings of the Eustachian tube).

High blood pressure in children is defined as a mean blood pressure at or above the 95th percentile for their age, sex, and height when measured multiple times over three or more visits.

#### Monitoring and Management of Sickle Cell Disease in French Guiana

After the announcement of the diagnosis of SCD by the pediatricians, subsequent consultations alternate between visits to the maternal and child protection centers and/or private physicians, who renew explanations, provide vaccinations, carry out surveillance, and in hospital specialized care. We have implemented prophylactic penicillin treatment for all sickle cell patients, comprehensive vaccination and education about regular spleen size measurement for sickle cell families. Hospital visits take place every 3 months in the first 2 years of life, and then at a frequency depending on the severity of the disease. At each visit, the parents’ ability to recognize events requiring an urgent consultation (fever higher than 38.5 C°, sudden pallor and/or asthenia, pain not responding to initial analgesic treatment, sudden increase of spleen volume, vomiting) is verified. Similarly, risk factors for vasoocclusive crisis (VOC) are regularly explained. Clinical examination looks for possible hepatosplenomegaly. The splenic overflow is noted in the health booklet, and parents are taught how to measure the spleen, so that they can recognize a sudden increase in its volume. The ears, nose, and throat (ENT) sphere is also examined for upper airway obstruction. The annual check-up varies according to the child’s age. A blood count with reticulocyte count, iron status, blood electrolytes and liver function tests are performed annually. From the age of 3, the chest X-ray and abdominal ultrasound are checked annually, and from the age of 6, the pelvic X-ray and cardiac ultrasound are checked. Ophthalmologic surveillance is performed from the age of 6 years in SC children and 10 years in SS children. Cerebral vascularization is systematically checked by TCD from the age of 12–18 months in homozygous children and those who are S-beta0 thalassemic. Before 2009, the frequency of TCD depended on the SCD standard of care of each specialized center. Since 2009, with the establishment of the sickle cell competence centers in French Guiana, TCD was performed annually for each homozygous sickle cell child within the framework of a standardized protocol in all 3 SCD specialized-care centers of French Guiana (Cayenne, Saint-Laurent-du-Maroni and Kourou).

In case of pathological velocities documented by TCD, the child is enrolled in an immediate transfusion exchange program for 2–3 months, followed by brain and cervical magnetic resonance imaging (MRI) with Doppler monitoring. When a stenosis is found, the exchange program is continued in the long term (undetermined duration, HLA typing in siblings and indication of bone marrow transplant in case of HLA compatible donor). When the imaging is normal, the program is continued for 1 year. If after 1 year, there is still no stenosis on imaging patient care depends on TCD velocities:

- TCD velocities are still pathological or borderline: exchanges are continued with annual TCD monitoring until normalization.
- TCD velocities are normalized: the child starts Hydroxyurea (HU) treatment up to the maximum tolerated dose with at least 2 months of overlap with the exchange program, with a TCD check every 3 months and MRI every year. Hydroxyurea is continued if examinations (Doppler and MRI) are normal, with quarterly TCD monitoring for 1 year and then annually. In case of pathological velocities, the exchange transfusion program is reinstated in the long term.

In case of pathological extracranial velocities on TCD, the child is enrolled in a transfusion exchange program. When these extracranial velocities are borderline normal, MRI is indicated because of the significant risk of stenosis. If there is a stenosis, a long-term exchange program is recommended. If there is no stenosis, the child is put on HU with quarterly TCD monitoring. The transfusion or to transfusion exchanges were identical in the three centers of French Guiana.

### Study Design

This was a multicenter retrospective cohort study, conducted at the Integrated Sickle Cell Center of French Guiana, a reference center based at Cayenne hospital. A single reviewer (JG) extracted all the records of all children with SCD, admitted to the pediatric units of the three hospitals of French Guiana (Cayenne, Saint-Laurent-du-Maroni and Kourou), born between 01/01/1992 and 12/31/2002, aged < 16 years at the time of inclusion. Patients were identified using the International Classification of Diseases (ICD 10) codes for sickle cell disease (D570, D571, D572) in electronic databases of the different pediatric departments of participating hospitals. The routine sickle cell testing such as newborn screening was implemented in 1992 in French Guiana. All children were diagnosed in the perinatal period. The confirmation of SCA was done using Hb electrophoresis at 6 months of age. Clinical, laboratory, and radiologic monitoring data were collected up to the age of 15 years and 3 months (pediatric age), or the date of the latest news. The failure event was clinical stroke. The biological recorded data were those of the last year of follow-up, reflecting baseline conditions (temporally separated from any acute or chronic event). For each patient, basic information and specific clinical complications were collected: age, gender, hemoglobin type, haplotype, alpha-thalassemia, severity and number of prior acute or chronic sickle cell specific complications (acute splenic or hepatic sequestration, acute chest syndrome, sickling related painful vasoocclusive crisis, neurologic events, severe infections, acute anemia, cholelithiasis), use of opioids for painful events, use and number of transfusions. These data were collected from computerized and/or paper patient records and were directly entered into an electronic form (eCRF) built using Clinsight Capture System^®^ software. The inclusion and data collection period extended from 02/01/2019 to 09/30/2019, and the database was frozen on December 31st, 2019. Only the first stroke admission was included in the analysis. Repeated admissions of the same patient with stroke were excluded.

### Statistical Analysis

Statistical data analysis was performed using Stata 12.0 (Stata Statistical Software: Release 12. College Station, TX: StataCorp LP). Categorical data were summarized as count (percentage) and quantitative data as median with 25th/75th percentiles (interquartile range, IQR), and compared using the χ^2^ test or Fisher’s exact test. Single failure survival analysis was performed and Kaplan-Meier curves were plotted. *P* < 0.05 was chosen as the statistical significance threshold. The exact date of entry of each subject was the birth date. The end of the follow-up period was the date of latest news (up to 15 years 3 months) or date of death (if applicable). The stroke date represented the failure event date if it occurred.

The estimated sample size needed to obtain a 20% difference between the incidence observed in the Freepong study and ours, with a 2-sided test, 90% power, and 5% alpha error, was 70 subjects in total.

### Ethical Approval

This study was approved by the « Comité d’Evaluation Ethique de l’Inserm (Number IRB00003888) and the database was declared at the Commission Nationale Informatique et Liberté (CNIL, Number 2200250 v 0). Patients were informed of the utilization of their data with an informative poster in the medical units concerned with SCD. All patients and relatives were personally informed and asked to give approval to participate in the stud. All data were collected, after certification of a written patient’s non-opposition. All underage participants had written informed consent provided on their behalf by their parent/legal guardian.

## Results

**FIGURE 1:**
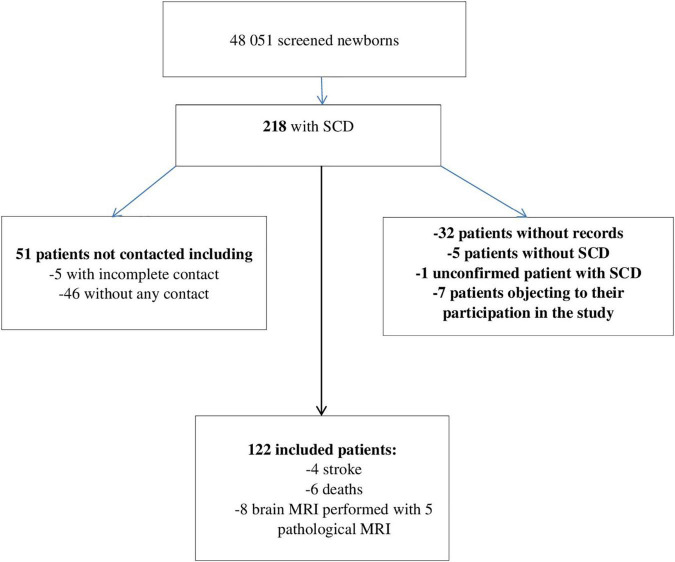
Study flow diagram. SCD, sickle cell disease; Stroke, cerebrovascular accidents; MRI, magnetic resonance imaging.

A total of 218 records were extracted 122 of which met the inclusion criteria, as shown in Figure 1. Our cohort comprised 56 females (46%), and 66 males (54%). There were 70 HbSS/Sβ0 (58%), 40 HbSC (33%), and 11 Sβ + thalassemia (9%). Of the 122 included patients, 60 lived in the urban Municipality of Cayenne, 49 in Western French Guiana and 11 patients lived in the Savanas. Among them, 21 (17.21%) were treated with HU. The number of emergency room admissions was significantly different between genotypes, with a higher number in SS/Sβ0 children, as expected (Table 1). Frequency of acute chest syndrome (ACS) and the level of lactate dehydrogenase (LDH) were significantly higher in patients with genotype SS/Sβ0 (Tables 2, 3). Among the 108 patients who performed a TCD, there were 17% abnormal, 25% conditional, and 58% normal TCD. For all of them, the cerebral MRI angiography was normal. A transfusion exchange program was offered to these 18 patients with pathological TCD. However, only 11 patients were able to benefit from it, for 12 months, after which the transfusion program was stopped. The TCD of the transfused patients was normal by the sixth month after the start of the exchange program. The remaining 7 patients were all treated with HU for the following reasons:

**TABLE 1:** Clinical characteristics of children with sickle cell disease, according to their genotype.

	SS/Sβ 0		SC		Sβ +		NS*	p
	*N* = 70	%	*N* = 40	%	*N* = 11	%	*N* = 1	(α = 5%)
**Gender**								0.269
Male	42	60	20	50	4	36		
Female	28	40	20	50	7	64	1	
**Hospital follow-up**								0.366
Cayenne	44	63	20	50	8	73		
Kourou	6	8	2	5	0	0		
Saint Laurent du Maroni	20	29	18	45	3	27	1	
**Haplotype**	*n* = 28		*n* = 10		*n* = 6			0
Benin/Benin	12	43	0	0	1	17		
Benin/Bantu	5	18	0	0	0	0		
Benin/atypical	4	14	0	0	0	0		
Bantu/Atypical	3	11	0	0	0	0		
Benin/Beta C allele	0	0	7	70	0	0		
Others	4	14	3	30	5	83		
**Number of hospitalizations**	*n* = 59		*n* = 33		*n* = 11			0
None	1	2	11	33	2	18		
1–4	15	25	17	52	4	36	1	
5–9	13	22	3	9	1	9		
10 or more	30	51	2	6	4	36		
**Number of emergency room visits**	*n* = 48		*n* = 27		*n* = 9			0.004
None	1	2	3	11	0	0		
1–4	5	10	12	44	1	11	1	
5–9	11	23	4	15	3	33		
10 or more	31	65	8	30	5	56		
**Alpha thalassemia**	*n* = 34		*n* = 11		*n* = 6			0.634
2/4 alleles	2	6	2	18	0	0		
3/4 alleles	11	32	2	18	2	33		
None	21	62	7	64	4	67		

*NS, not specified.

**TABLE 2:** Distribution of clinical history by genotype.

	Genotypes										*p*
	SS/Sβ 0			SC			Sβ +			NS (*N* = 1)	(α = 5%)
	*N*	Presence	%	*N*	Presence	%	*N*	Presence	%	Presence	
ACS (*n* = 122)	70	20	29	40	2	5	11	2	18	0	0.006
AHT (*n* = 90)	54	1	2	26	0	0	10	1	10		0.292
Infant asthma (*n* = 15)	12	3	25	2	0	0				0	1
Asthma (*n* = 99)	57	9	16	31	6	19	11	0	0		0.33
Hypertrophy of adenoids (*n* = 25)	18	7	39	2	0	0	4	1	25	1	0.798
Enlarged tonsils (*n* = 77)	52	7	13	18	1	6	6	0	0	0	0.835

*NS, not specified; ACS, acute chest syndrome; AHT, arterial hypertension.*

**TABLE 3:** Comparative incidence of first CVA in children with SCA (SS and β0 thalassemia).

Study by	Ohene-Frempong et al.	Our study		Lagunju et al.
Number of patients	*n* = 4,082	*n* = 122		*n* = 104
Genotyes	67.8% SS	58% SS&S-β0 thal		100% Hb SS
	22.1% SC	33% SC		
	5.2% S-β + thal	9% β + thal		
	4.9% S-β0 thal			
Prevalence of stroke (%)	6.44	6.4		2.3
Incidence rate (per 100 patient-years)	0.69	0.31	(95% CI: 0.1–0.97)	0.27

*CVA, cerebrovascular accident; SCA, Sickle cell anemia; Thal, thalassemia.*

- alloimmunization and difficulty crossmatching units in 4 patients
- refusal of transfusion programs in the other 3 patients

**FIGURE 2:**
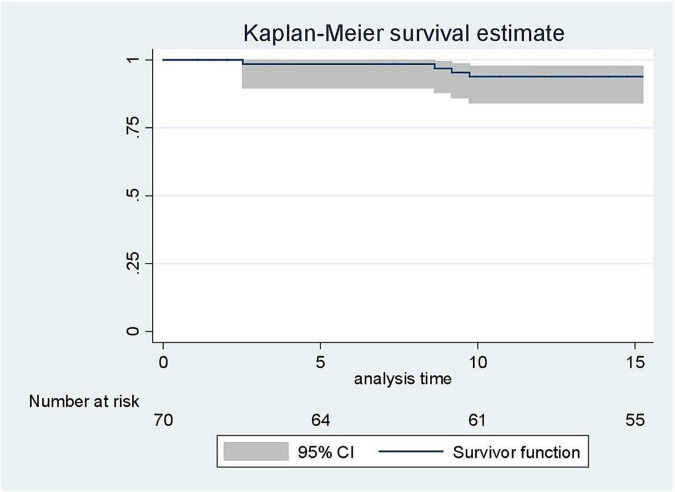
Kaplan-Meier curve on the estimate of the occurrence of clinical stroke before 15 years 3 months.

A total of 4 patients had a diagnosis of stroke. Three of these patients experienced a ischemic stroke at a mean age of 6.9 years, and one a hemorrhagic stroke at 9.2 years of age. The cumulative incidence rate of ischemic stroke among SS/Sβ0 children was 3.1 (95% CI: 1.0–9.7) per 1,000 patient-years (Figure 2), and the risk of clinical stroke by age 15 years and 3 months was 6,4%. The incidence of hemorrhagic stroke was 1.1 (95% CI: 0.1–7.4) per 1,000 patients-years. All strokes occurred only in children with SS/SB0 genotypes. Risk factors such as lack of alpha-thalassemia, high blood pressure, asthma, and enlarged adenoids were not significantly different between genotypes (Tables 1, 2). 15 patients in this study had moderate splenomegaly. There was no difference in splenomegaly according to genotype. Only 8 patients benefited from a magnetic resonance imaging (MRI) of the brain. Four silent brain infarctions (SBI) were diagnosed (including 2 prior to ischemic stroke), two internal carotid artery stenoses, as well as brain images corresponding to migraine lesions in one patient. There were no cases of transient ischemic attack (TIA) in our study.

Six deaths occurred during the study period, all in patients with SS/Sβ0 SCD except one in a patient with an unknown genotype. The sex ratio was 1. Three patients were followed in Cayenne, and the three others in Saint Laurent du Maroni. Death occurred at a mean age of 4.9 years [range: 9 months–14.75 years]. Two deaths were attributed to severe acute anemia (Hb level 2 and 3 g/dL), two other deaths occurred at home, one in a febrile context, without further information. One death was attributable to an acute chest syndrome complicated by coma, without brain imaging performed. The last death was secondary to post-anesthesia cardiac-respiratory arrest following phlegmon surgery, the injected brain scan was normal. None of our patients benefited from bone marrow transplantation (BMT).

## Discussion

The incidence of clinical stroke in children with SS/Sβ0 was low although comparable to previous studies (5, 6, 19). This seems to be explained by a combination of different factors, among which we believe the implementation of systematic screening for cerebral vasculopathy by TCD has played a major role since 2009. The comparison with Ohene-Frempong et al. study (5) (Table 3) shows an incidence rate close to ours. In addition, our prevalence, although lower, is close to that of Ohene-Frempong et al. study (5). According to the literature, stroke rates vary with sickle cell genotype. Children with HbSS or HbSβ^0^ thalassemia have a higher risk of stroke compared with SC and HbSβ + thalassemia. In our study, no patient with SC or Sβ + thalassemia genotypes experienced stroke. However, the proportion of SC and HbSβ + patients was high compared with the Frempong cohort. The protective effect of alpha-thalassemia against the development of stroke is well established in the literature. The high rates of subjects with an alpha-thalassemic trait (37.2%) could also explain this low stroke incidence rate. The high Hb F in SSS/Sβ0 could be explained by the young age of our study population. Other factors described in the literature could not be tested because of the retrospective nature of our study. The impact of early TCD screening and intensive therapy on cerebral vasculopathy is now globally recognized (20–22). Thus, our protocol follows the international recommendations to perform an annual TCD from the age of 18 months until the age of 16–18 years (20–22). A limited number of patients under study benefited from a brain MRI. This examination, introduced in our protocol in 2009, has become more accessible since 2015, with the acquisition by each of the three hospitals of an MRI scanner and the presence of another MRI scanner within a private radiology department in Cayenne. For this reason, very few MRIs have been performed. Thus, in a follow-up cohort of children screened for SCA since birth, the systematic detection of pathological TCD and the implementation of an early transfusion program made it possible to reduce the cumulative risk of stroke before age 18 years to 1.9 vs. 11% in the natural history of the disease (10). This is what we have been trying to do since the introduction of TCD. We recognize that stopping the transfusion program might result in a risk of observing another pathological TCD, or even stroke (23–25). However, the transfusion protocol is cumbersome and sometimes inaccessible for some patients. This is why we have implemented this lighter protocol, which allows for the use of HU after 1 year of exchange transfusion (26).

A study in Nigeria showed that HU significantly reduced TCD velocities in children with SCD and high TCD velocities, with a drastic reduction in primary stroke incidence (27). HU is recognized as a potential alternative for primary stroke prevention in low- and middle-income countries (27–29). None of our patients benefited from BMT. However, the place of BMT as a primary prevention strategy was evaluated by the DREPAGREFFE study, which compares the outcome of cerebral vasculopathy following geno-identical allogeneic transplantation vs. chronic transfusion program (30). In French Guiana, the health system is that of France with free access to care and treatment. The neonatal screening for SCD has been generalized since 1992 (13). And the routine TCD screening with indefinite chronic red blood cell transfusion for children with abnormal TCD as standard of care has been implemented since 2009. Our practices regarding the indication and prescription of brain MRI have therefore improved with the increase in the availability of MRI in French Guiana. Currently, in the 3 hospitals, the protocol for the management of cerebral vasculopathy with annual TCD and angio-MRI in case of pathological TCD (and HLA typing in siblings and indication of bone marrow transplant in case of HLA compatible donor) is scrupulously applied. This has led to an improvement in the management of this serious complication of SCD. The hypothesis that the number of strokes could have been influenced by the number of lost to follow-up is unlikely. Indeed, the occurrence of a clinical stroke implies hospitalization in one of the three hospitals of French Guiana, unless an immediate death occurs at home. In our study, over 10 years, there were 6 deaths in children. In the identified causes of death linked to SCD in France at any age, stroke was the most important cause identified: 34 cases out of 412 deaths (31). The small number of deaths that occurred in our study could be related to the low incidence of stroke. The frequency of silent strokes cannot be discussed in view of the low proportion of children screened with brain MRIs. Screening for silent strokes in children should be improved. Although there are no clear guidelines, sickle cell patients and their families may benefit from training to recognize the signs of TIA, which are sometimes inconspicuous, and to get them to consult urgently even if the symptoms disappeared. A therapeutic education program for sickle cell patients with a dedicated nurse was started in January 2020 at the Integrated Sickle Cell Center at Cayenne hospital.

Our study has some biases and limitations. Our major limitation is the low number of children who had a stroke in the cohort, which makes the identification of risk factors associated with stroke difficult. However, we have not yet collected data on the incidence of stroke in the post-TCD era. In this retrospective study, with many patients lost to follow-up and missing data-the older medical records were sometimes not exhaustive. The beginning of cerebrovascular screening overlapped with a period of our study, and this may have reduced the number of strokes found. However, since the risk of stroke occurred mainly before 9 years, the impact of this bias is difficult to estimate. A recent study carried out in the United States following the STOP and STOP 2 trials showed that the appearance of a first ischemic stroke was due to a failure in the monitoring and treatment of patients in 63% of cases (32).

Despite these limitations, this multicenter study made it possible to estimate the incidence and risk factors of clinical stroke in children with SCD in French Guiana. These results will be taken into account to improve the screening and the primary and secondary prevention of cerebral vasculopathy in children with SCD in French Guiana.

## Conclusion

The risk of clinical stroke in children with SS/Sβ0 SCD before the age of 15 years and 3 months was low. This was probably explained by the implementation of routine TCD screening with indefinite CRCT for children with abnormal TCD. Additional opportunities for ischemic stroke prevention through the recent implementation of therapeutic patient education program may further reduce the incidence of this complication and will be evaluated.

## Data Availability Statement

The raw data supporting the conclusions of this article will be made available by the authors, without undue reservation.

## Ethics Statement

The studies involving human participants were reviewed and approved by Comité d’Evaluation Ethique de l’Inserm (Number IRB00003888). Written informed consent to participate in this study was provided by the participants’ legal guardian/next of kin.

## Author Contributions

All authors listed have made a substantial, direct, and intellectual contribution to the work, and approved it for publication.

## Conflict of Interest

The authors declare that the research was conducted in the absence of any commercial or financial relationships that could be construed as a potential conflict of interest.

## Publisher’s Note

All claims expressed in this article are solely those of the authors and do not necessarily represent those of their affiliated organizations, or those of the publisher, the editors and the reviewers. Any product that may be evaluated in this article, or claim that may be made by its manufacturer, is not guaranteed or endorsed by the publisher.

## References

[1] Azar S Wong TE. Sickle cell disease: a brief update. *Med Clin North Am.* (2017) 101:375–93. 10.1016/j.mcna.2016.09.009 28189177

[2] McGann PT Nero AC Ware RE. Ware. Current management of sickle cell anemia. *Cold Spring Harb Perspect Med.* (2013) 3:a011817. 10.1101/cshperspect.a011817 PMC3721270 23709685

[3] Makani J Williams TN Marsh K. Sickle cell disease in Africa: burden and research priorities. *Ann Trop Med Parasitol.* (2007) 101:3–14. 10.1179/136485907X154638 17244405 PMC5612390

[4] Guilliams KP Fields ME Dowling MM. Advances in understanding ischemic stroke physiology and the impact of vasculopathy in children with sickle cell disease. *Stroke.* (2019) 50:266–73. 10.1161/STROKEAHA.118.020482 30661504 PMC6385587

[5] Ohene-Frempong K Weiner SJ Sleeper LA Miller ST Embury S Moohr JW Cerebrovascular accidents in sickle cell disease: rates and risk factors. *Blood.* (1998) 91:288–94. 9414296

[6] Connes P Verlhac S Bernaudin F. Advances in understanding the pathogenesis of cerebrovascular vasculopathy in sickle cell anaemia. *Br J Haematol.* (2013) 161:484–98. 10.1111/bjh.12300 23496688

[7] Belisário AR Silva CM Velloso-Rodrigues C Viana MB. Genetic, laboratory and clinical risk factors in the development of overt ischemic stroke in children with sickle cell disease. *Hematol Transfus Cell Ther.* (2018) 40:166–81. 10.1016/j.bjhh.2017.08.008 30057991 PMC6003005

[8] Quinn CT McKinstry RC Dowling MM Ball WS Kraut MA Casella JF Acute silent cerebral ischemic events in children with sickle cell anemia. *JAMA Neurol.* (2013) 70:58–65. 10.1001/jamaneurol.2013.576 23108767 PMC3677221

[9] Adams RJ McKie VC Hsu L Files B Vichinsky E Pegelow C Prevention of a first stroke by transfusions in children with sickle cell anemia and abnormal results on transcranial Doppler ultrasonography. *N Engl J Med.* (1998) 339:5–11. 10.1056/NEJM199807023390102 9647873

[10] Bernaudin F Verlhac S Arnaud C Kamdem A Chevret S Hau I Impact of early transcranial Doppler screening and intensive therapy on cerebral vasculopathy outcome in a newborn sickle cell anemia cohort. *Blood.* (2011) 117:1130–40. 10.1182/blood-2010-06-293514 21068435

[11] Enninful-Eghan H Moore RH Ichord R Smith-Whitley K Kwiatkowski JL. Transcranial Doppler ultrasonography and prophylactic transfusion program is effective in preventing overt stroke in children with sickle cell disease. *J Pediatr.* (2010) 157:479–84. 10.1016/j.jpeds.2010.03.007 20434165 PMC2931594

[12] Brewin J Kaya B Chakravorty S. How I manage sickle cell patients with high transcranial doppler results. *Br J Haematol.* (2017) 179:377–88. 10.1111/bjh.14850 28771666

[13] Etienne-Julan M Elana G Loko G Elenga N Vaz T Muszlak M. La drépanocytose dans les départements français d’outre-mer (Antilles, Guyane, la Réunion, Mayotte): données descriptives et organisation de la prise en charge. *Bull Épidémiol Hebd.* (2012) 27–28:322–5.

[14] Simonnet C Elanga N Joly P Vaz T Nacher M. Genetic modulators of sickle cell disease in French Guiana: markers of the slave trade. *Am J Hum Biol.* (2016) 28:811–6. 10.1002/ajhb.22871 27251090

[15] Knight-Madden J Lee K Elana G Elenga N Marcheco-Teruel B Keshi N Newborn screening for sickle cell disease in the caribbean: an update of the present situation and of the disease prevalence. *Int J Neonatal Screen.* (2019) 5:5. 10.3390/ijns5010005 33072965 PMC7510201

[16] Elenga N Cuadro E Martin É Cohen-Addad N Basset T. Associated factors of acute chest syndrome in children with sickle cell disease in French Guiana. *Int J Pediatrics.* (2014) 2014:213681. 10.1155/2014/213681 24678322 PMC3942098

[17] Wijdicks EF Sheth KN Carter BS Greer DM Kasner SE Kimberly WT Recommendations for the management of cerebral and cerebellar infarction with swelling: a statement for healthcare professionals from the American Heart Association/American Stroke Association. *Stroke.* (2014) 45:1222–38. 10.1161/01.str.0000441965.15164.d6 24481970

[18] Sacco RL Kasner SE Broderick JP Caplan LR Connors JJ Culebras A An updated definition of stroke for the 21st century: a statement for healthcare professionals from the American Heart Association/American Stroke Association. *Stroke.* (2013) 44:2064–89. 23652265 10.1161/STR.0b013e318296aeca PMC11078537

[19] Hirtz D Kirkham FJ. Sickle cell disease and stroke. *Pediatr Neurol.* (2019) 95:34–41. 30948147 10.1016/j.pediatrneurol.2019.02.018

[20] Adams RJ McKie VC Brambilla D Carl E Gallagher D Nichols FT Stroke prevention trial in sickle cell anemia. *Control Clin Trials.* (1998) 19:110–29. 9492971 10.1016/s0197-2456(97)00099-8

[21] Adams RJ Brambilla DJ Granger S Gallagher D Vichinsky E Abboud MR Stroke and conversion to high risk in children screened with transcranial Doppler ultrasound during the STOP study. *Blood.* (2004) 103:3689–94. 10.1182/blood-2003-08-2733 14751925

[22] Lee MT Piomelli S Granger S Miller ST Harkness S Brambilla DJ Stroke prevention trial in sickle cell anemia (STOP): extended follow-up and final results. *Blood.* (2006) 108:847–52. 10.1182/blood-2005-10-009506 16861341 PMC1895848

[23] Adams RJ Brambilla D. Optimizing primary stroke prevention in sickle cell anemia (STOP 2) trial investigators. discontinuing prophylactic transfusions used to prevent stroke in sickle cell disease. *N Engl J Med.* (2005) 353:2769–78. 10.1056/NEJMoa050460 16382063

[24] Abboud MR Yim E Musallam KM Adams RJ. STOP II study investigators. discontinuing prophylactic transfusions increases the risk of silent brain infarction in children with sickle cell disease: data from STOP II. *Blood.* (2011) 118:894–8. 10.1182/blood-2010-12-326298 21633086 PMC3148169

[25] DeBaun MR Gordon M McKinstry RC Noetzel MJ White DA Sarnaik SA Controlled trial of transfusions for silent cerebral infarcts in sickle cell anemia. *N Engl J Med.* (2014) 371:699–710. 10.1056/NEJMoa1401731 25140956 PMC4195437

[26] Ware RE Davis BR Schultz WH Brown RC Aygun B Sarnaik S Hydroxycarbamide versus chronic transfusion for maintenance of transcranial doppler flow velocities in children with sickle cell anaemia-TCD With Transfusions Changing to Hydroxyurea (TWiTCH): a multicentre, open-label, phase 3, non-inferiority trial. *Lancet.* (2016) 387:661–70. 10.1016/S0140-6736(15)01041-7 26670617 PMC5724392

[27] Lagunju I Brown BJ Oyinlade AO Asinobi A Ibeh J Esione A Annual stroke incidence in Nigerian children with sickle cell disease and elevated TCD velocities treated with hydroxyurea. *Pediatr Blood Cancer.* (2019) 66:e27252. 10.1002/pbc.27252 29797633

[28] Adegoke SA Macedo-Campos RS Braga JAP Figueiredo MS Silva GS. Changes in transcranial Doppler flow velocities in children with sickle cell disease: the impact of hydroxyurea therapy. *J Stroke Cerebrovasc Dis.* (2018) 27:425–31. 10.1016/j.jstrokecerebrovasdis.2017.09.020 29056404

[29] Hankins JS McCarville MB Rankine-Mullings A Reid ME Lobo CL Moura PG Prevention of conversion to abnormal transcranial Doppler with hydroxyurea in sickle cell anemia: a Phase III international randomized clinical trial. *Am J Hematol.* (2015) 90:1099–105. 10.1002/ajh.24198 26414435 PMC4715740

[30] Chevret S Verlhac S Ducros-Miralles E Dalle JH de Latour RP de Montalembert M Design of the DREPAGREFFE trial: a prospective controlled multicenter study evaluating the benefit of genoidentical hematopoietic stem cell transplantation over chronic transfusion in sickle cell anemia children detected to be at risk of stroke by transcranial Doppler (NCT 01340404). *Contemp Clin Trials.* (2017) 62:91–104. 10.1016/j.cct.2017.08.008 28821470

[31] Gomes E Castetbon K Goulet V. Mortalité liée à la drépanocytose en France: âge de décès et causes associées (1979-2010). *Bull Épidémiol Hebd.* (2015) 8:142–50.

[32] Kwiatkowski JL Voeks JH Kanter J Fullerton HJ Debenham E Brown L Ischemic stroke in children and young adults with sickle cell disease in the post-STOP era. *Am J Hematol.* (2019) 94:1335–43. 10.1002/ajh.25635 31489983

